# Dissociation between sublingual and gut microcirculation in the response to a fluid challenge in postoperative patients with abdominal sepsis

**DOI:** 10.1186/s13613-014-0039-3

**Published:** 2014-12-04

**Authors:** Vanina Siham Kanoore Edul, Can Ince, Noelia Navarro, Luciana Previgliano, Alejandro Risso-Vazquez, Paolo Nahuel Rubatto, Arnaldo Dubin

**Affiliations:** 1 Academic Medical Center, Department of Translational Physiology, Amsterdam, Netherlands; 2 Facultad de Ciencias Médicas, Universidad Nacional de La Plata, Cátedra de Farmacología Aplicada, Calle 42 N° 577 (1900), La Plata, Argentina; 3 Sanatorio Otamendi y Miroli, Servicio de Terapia Intensiva, Buenos Aires, Argentina

**Keywords:** Microcirculation, Tissue perfusion, Fluid challenge, Septic shock, Intestine, Sublingual, Abdominal surgery

## Abstract

**Background:**

This study was performed to compare intestinal and sublingual microcirculation and their response to a fluid challenge.

**Methods:**

Twenty-two septic patients in the first postoperative day of an intestinal surgery, in which an ostomy had been constructed, were evaluated both before and 20 min after a challenge of 10 mL/kg of 6% hydroxyethylstarch 130/0.4. We measured systemic hemodynamics and sublingual and intestinal microcirculation. Correlations between variables were determined through the Pearson test.

**Results:**

Fluid administration increased the cardiac index (2.6 ± 0.5 vs. 3.3 ± 1.0 L/min/m^2^, *P* < 0.01) and mean arterial blood pressure (68 ± 11 vs. 82 ± 12 mm Hg, *P* < 0.0001). The sublingual but not the intestinal red blood cell (RBC) velocity increased (912 ± 270 vs. 1,064 ± 200 μm/s, *P* < 0.002 and 679 ± 379 vs. 747 ± 419 μm/s, *P* = 0.12, respectively). The sublingual and intestinal perfused vascular density (PVD) did not change significantly (15.2 ± 2.9 vs. 16.1 ± 1.2 mm/mm^2^ and 12.3 ± 6.7 vs. 13.0 ± 6.7 mm/mm^2^). We found no correlation between the basal sublingual and intestinal RBC velocities or between their changes in response to the fluid challenge. The individual changes in sublingual RBC velocity correlated with those in cardiac index and basal RBC velocity. Individual changes in intestinal RBC velocity did not correlate with either the cardiac index modifications or the basal RBC velocity. The same pattern was observed with the sublingual and the intestinal PVDs. The sublingual RBC velocities and PVDs were similar between survivors and nonsurvivors. But the intestinal RBC velocities and PVDs were lower in nonsurvivors.

**Conclusions:**

In this series of postoperative septic patients, we found a dissociation between sublingual and intestinal microcirculation. The improvement in the sublingual microcirculation after fluid challenge was dependent on the basal state and the increase in cardiac output. In contrast, the intestinal microcirculation behaved as an isolated territory.

## Background

Patients with septic shock characteristically exhibit different kinds of sublingual microcirculatory abnormalities. The main microvascular alterations consist in a decrease in the proportion of perfused vessels and the perfused vascular density (PVD) along with an increase in vessel heterogeneity [1],[2]. The presence of these abnormalities identifies the patients with worse outcomes [1]-[5]. Moreover, some therapeutic approaches may improve these derangements regardless of changes in systemic cardiovascular effects [6],[7]. Accordingly, the presence of sublingual microvascular alterations may help to identify patients eligible for fluid therapy [8].

Most of the studies have been focussed on the sublingual microcirculation because that location is easily accessible. In addition, the tongue has the same embryologic origin as the intestine and is richly vascularized by a branch of the external carotid artery. Nevertheless, controversies have arisen about the relative behavior of other microvascular beds. A particular concern is if sublingual microcirculation reflects other territories, such as the intestinal villi. If not, gut ischemia might be present even when the sublingual mucosa is well perfused. The relevance of this issue lies in the possibility that the persistence of villi hypoperfusion may lead to alterations in the barrier function with the subsequent systemic translocation of bacteria and their products, one conceivable mechanism of multiple organ failure [9].

Experimental research on animals has shown that the response of sublingual microcirculation to volume expansion may differ from that of the gut. In a sheep model, fluid resuscitation normalized sublingual microcirculation, but the intestinal villi remained hypoperfused [10]. In another animal study, fluid resuscitation improved both areas, but the intestinal PVD did not reach basal values [11]. In contrast, in an experimental cholangitis study, comparable sublingual and gut alterations were reported [12]. The only clinical investigation on this question indicated a lack of correlation between both compartments in postoperative septic patients [13].

Our hypothesis was that the sublingual and intestinal microcirculations have different behaviors. Therefore, our goal was to describe the correlation of those two territories both under basal conditions and in response to a fluid challenge.

## Methods

### Ethical approval and informed consent

The study was approved by the Comité de Ética en Investigaciones Biomédicas, Sanatorio Otamendi y Miroli. Written consent was obtained from the next of kin of all patients admitted to the study.

### Design

This study is a prospective, observational study.

### Setting

The study was conducted in a teaching intensive care unit (ICU) in a university-affiliated hospital.

### Patients

We studied 22 adult patients (i.e., greater than 21 years of age) fulfilling the criteria of severe sepsis [14] during the first postoperative day of an emergency abdominal procedure. In all cases, the surgical finding was an intra-abdominal source of sepsis, which a clinical pattern required the construction of an intestinal ostomy - in 7 of the cases an ileostomy, while in the remaining 15 patients a colostomy. Each patient had a central venous line placed in the superior vena cava and an arterial line. The latter line was connected to a system for continuous cardiac output monitoring, based on pulse-contour analysis (Vigileo-FloTrac system, Edwards Lifesciences, Irvine, CA, USA). Patients were included during the 6-h period after surgery, if according to the attending physicians' criteria, they had clinical or laboratory findings of hypoperfusion that required a fluid challenge. These manifestations included a heart rate >100 beats/min, a urine output <0.5 mL/kg/h, a mean blood pressure ≤65 mm Hg, a central venous oxygen saturation <70%, a central venous arterial PCO_2_ > 6 mm Hg, hyperlactatemia ≥2.2 mmol/L, and a cardiac index <2.2 L/min/m^2^.

### Measurements and derived calculations

On the day of measurements, the demographic data (e.g., age, gender) were recorded and the Acute-Physiology and Chronic-Health Evaluation II (APACHE II) [15] and Sepsis-related Organ Failure Assessment (SOFA) scores [16] calculated. The ICU and hospital mortalities, the length of stay, the use of vasopressors, and the fluid balance were also registered. We measured the heart rate, the arterial and central venous pressures, and the cardiac output. The intra-abdominal pressure was measured by means of a urinary catheter after the instillation of 25 mL of saline solution. All pressure measurements were performed at the end of expiration. Zero pressure was referenced to atmospheric pressure at the mid-axillary line. Abdominal perfusion pressure was calculated as the difference between the mean arterial and intra-abdominal pressures. Arterial and central venous blood samples were analyzed for gases, hemoglobin, and oxygen saturation, and arterial lactate levels were also measured.

### Microvideoscopic measurements and analysis

The microcirculatory network was evaluated in the sublingual and the intestinal mucosa by means of a sidestream dark field (SDF) imaging device (Microscan, MicroVision Medical, Amsterdam, Netherlands) [17]. Different precautions were taken and steps followed to obtain images of adequate quality and to insure satisfactory reproducibility. After gentle removal of saliva or secretions by isotonic-saline-drenched gauze, steady images of at least 20 s were obtained while avoiding pressure artifacts through the use of a portable computer and an analog-to-digital video converter (ADVC110, Canopus Co., San Jose, CA, USA). The videos were recorded from five different areas in each territory. For the evaluation of the intestinal microcirculation, the device was inserted approximately 6 cm into the intestine through the stoma. Video clips were stored as AVI files to allow computerized frame-by-frame image analysis.

Video-image analysis was performed blindly by well-trained researchers. Adequate focus and contrast adjustment were verified and images of poor quality discarded. The entire sequence was used to characterize the semiquantitative characteristics of the microvascular flow and particularly the presence of stopped or intermittent flow.

We used an image-analysis software developed for the SDF video images [18] to determine the total vascular density. An analysis based on semiquantitative criteria that distinguished between no flow (0), intermittent flow (1), sluggish flow (2), and continuous flow (3) was performed on individual vessels [19]. The overall score, called the microvascular flow index (MFI), is the average of the individual values. Quantitative red blood cell (RBC) velocity was determined through the use of space-time diagrams [18]. We also calculated the proportion of perfused vessels and the PVD (i.e., the total vascular density multiplied by the fraction of perfused vessels). The heterogeneity-flow index was calculated as the highest MFI minus the lowest MFI divided by the mean MFI [20]. Flow heterogeneity was also evaluated as the coefficient of variation in the RBC velocities. The analysis was restricted to vessels with diameters <25 μm, whereas the vessels of higher diameter were assessed only for ruling out compression artifacts.

We also measured the gradient between the central and second finger pad temperature (ΔT°) as an estimate of the extent of skin perfusion. The ambient temperature was kept at 25°C.

### Procedure

After basal measurements, 10 mL/kg of 6% hydroxyethylstarch 130/0.4 in 0.9% NaCl (Voluven, Fresenius Kabi, Bad Homburg, Germany) was infused in 20 min. Measurements were repeated after the fluid challenge. We used only norepinephrine as a vasopressor and did not modify the dosage during the fluid challenge. On the following day, measurements were performed again in the patients that still remained alive in the ICU.

### Data analysis

According to previous results [2], we calculated that 22 patients were needed to show an increase of 20% in sublingual PVD, in response to the fluid challenge, with a power of 80% and a certainty of 95%. The normal distribution of the data was assessed by the Shapiro-Wilk test. The data are expressed as the mean ± SD. The differences between variables before and after volume expansion were evaluated by the paired Student *t* test. Correlations were determined through the Pearson test. We considered a *P* < 0.05 as statistically significant.

## Results

Table 1 summarizes the epidemiologic and clinical characteristics of the patients. The indications for the abdominal surgery were intestinal perforation (*n* = 8), intestinal ischemia (*n* = 5), intra-abdominal abscess (*n* = 3), dehiscence of previous anastomosis (*n* = 3), sigmoid volvulus (*n* = 2), and frozen pelvis (*n* = 1).

**Table 1:** **Epidemiological and clinical characteristics of the patients (*n*** **= 22)**

**Variable**	**Results**
Gender, male (*n*, %)	10 (45)
Age (years)	71 ± 16
APACHE II score	20 ± 8
SOFA score	7 ± 3
Use of norepinephrine	
Number of patients (*n*, %)	18 (82)
Dosage (μg/kg/min)	0.30 ± 0.23
Mechanical ventilation (*n*, %)	21 (95)
Tidal volume (mL/kg)	6.1 ± 1.3
Fluid intake in the first day (mL/24 h)	4,513 ± 2,063
Urine output in the first day (mL/24 h)	1,241 ± 836
Fluid balance in the first day (mL/24 h)	3,273 ± 2,110
ICU length of stay (days)	7 ± 5
Hospital length of stay (days)	16 ± 12
ICU mortality (*n*, %)	11 (50)
Hospital mortality (*n*, %)	11 (50)

The criteria for the fluid challenge were oliguria (*n* = 11); hyperlactatemia (*n* = 9); low central venous oxygen saturation (*n* = 8); high central venous arterial PCO_2_, tachycardia, and arterial hypotension (*n* =7); and low cardiac index (*n* = 6). Each patient had more than one indication for the volume expansion, and all hypotensive patients received norepinephrine.

### Macro- and microcirculatory responses to fluid challenge

**Figure 1:**
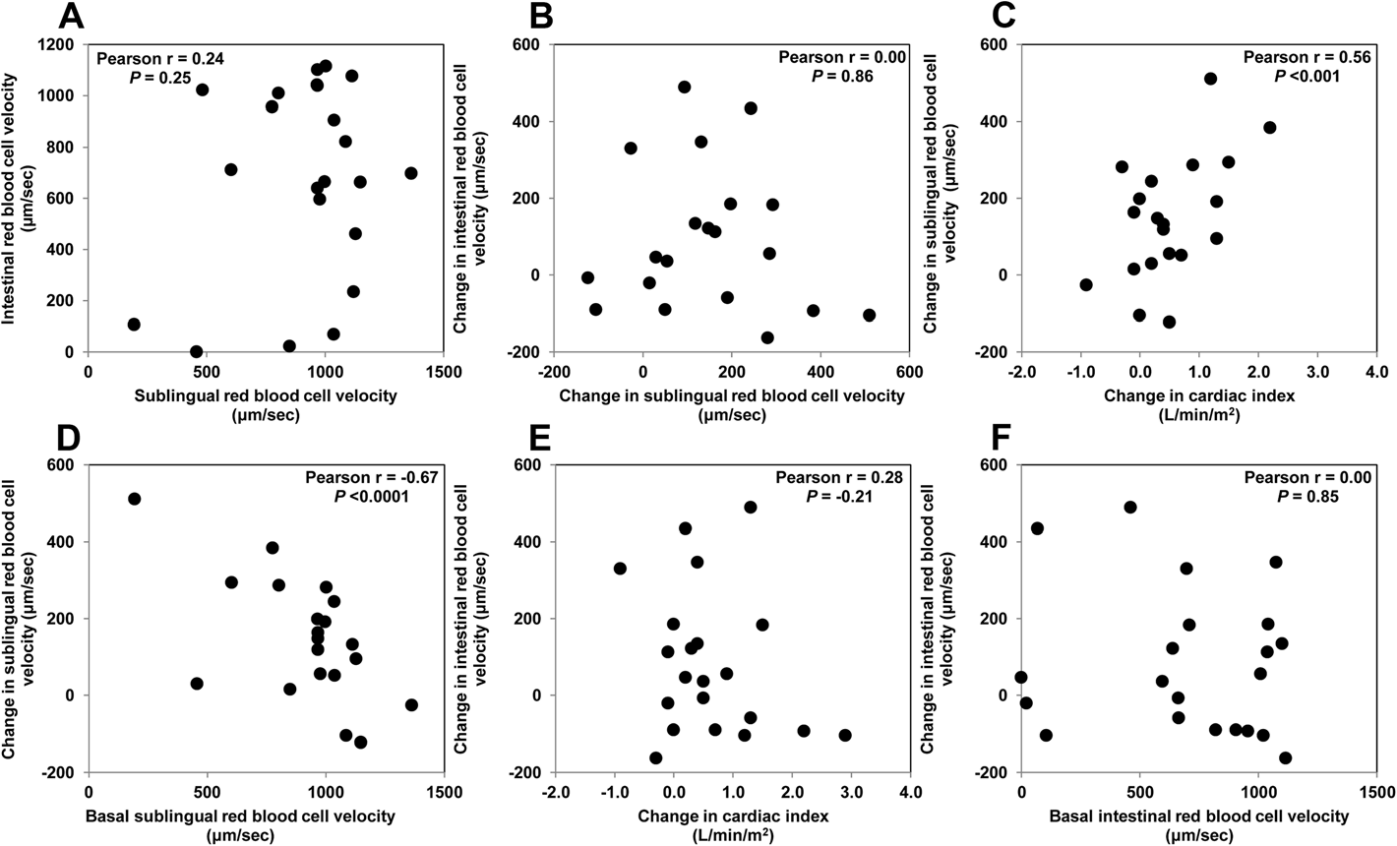
**Red blood cell (RBC) velocity. (A)** Correlation between basal sublingual and intestinal RBC velocity. **(B)** Correlation between the changes in sublingual and intestinal RBC velocities in response to the fluid challenge. **(C)** Correlation between the changes in cardiac index and sublingual RBC velocity in response to the fluid challenge. **(D)** Correlation between the changes in sublingual RBC velocity in response to the fluid challenge and the basal sublingual RBC velocity. **(E)** Correlation between the changes in cardiac index and intestinal RBC velocity in response to the fluid challenge. **(F)** Correlation between the changes in intestinal RBC velocity in response to the fluid challenge and the basal intestinal RBC velocity.

**Figure 2:**
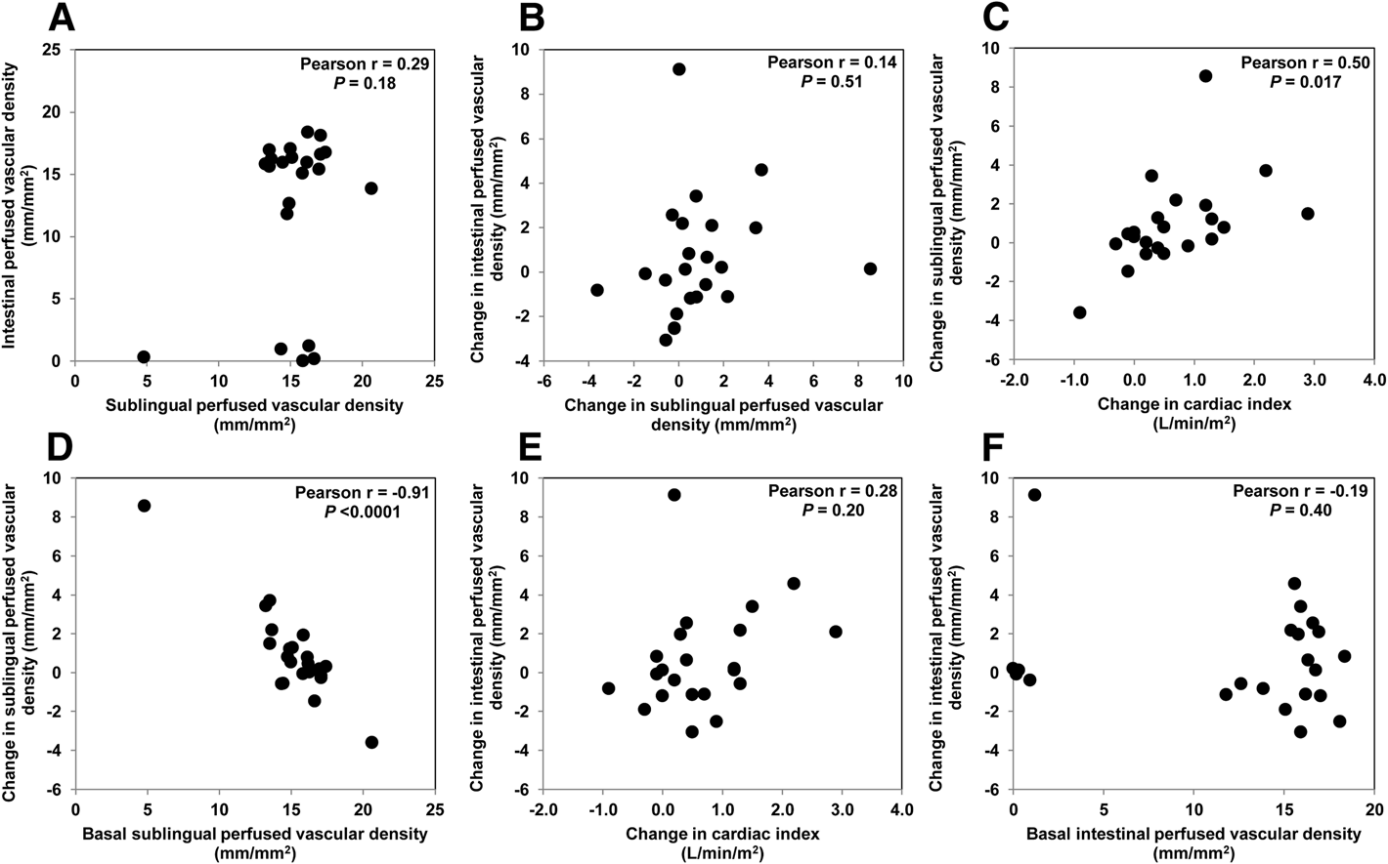
**Perfused vascular density (PVD). (A)** Correlation between basal sublingual and intestinal PVD. **(B)** Correlation between the changes in sublingual and intestinal PVD in response to the fluid challenge. **(C)** Correlation between the changes in cardiac index and sublingual PVD in response to the fluid challenge. **(D)** Correlation between the changes in sublingual PVD in response to the fluid challenge and the basal sublingual PVD. **(E)** Correlation between the changes in cardiac index and intestinal PVD in response to the fluid challenge. **(F)** Correlation between the changes in intestinal PVD in response to the fluid challenge and the basal intestinal PVD.

Table 2 displays systemic cardiovascular and tissue perfusion variables, before and after the fluid challenge in the entire group. The central venous, mean arterial, and abdominal perfusion pressures and the cardiac index all increased. The arterial lactate levels and the ΔT° did not change. Sublingual microcirculation showed a tendency to improve that only reached statistical significance with the RBC velocity and its corresponding coefficient of variation (Table 2). Nevertheless, a wide variation occurred in the individual responses to fluid challenge. The individual changes in the sublingual RBC velocity and PVD correlated with those in the cardiac index and with the respective basal values (Figures 1 and 2). Accordingly, in 17 fluid-responder patients (i.e., with a ≥15% increase in cardiac index), the sublingual PVD increased from 14.5 ± 2.9 to 16.0 ± 1.3 mm/mm^2^ (*P* < 0.02). Most of the other sublingual microvascular variables evidenced a similar pattern (Additional file 1: Figures S1, S2, S3). In contrast, the intestinal RBC velocity and PVD did not improve in the entire group of patients (Table 2), and neither of the individual changes in the two parameters were correlated with either the modifications in the cardiac index or the respective basal values. A similar result occurred with most of the other intestinal microvascular parameters (Additional file 1: Figures S1, S2, S3). The sublingual and intestinal PVD and RBC velocity showed no correlation with the cardiac index at baseline (*r* = −0.22, *P* = 0.35; *r* = −0.19, *P* = 0.40; *r* = −0.05; *P* = 0.83; and *r* = 0.22, *P* = 0.33, respectively).

**Table 2:** Systemic cardiovascular variables and tissue perfusion parameters before and after the fluid challenge

	**Before**	**After**	***P***
Heart rate (beats/min)	87 ± 27	86 ± 23	0.45
Mean arterial blood pressure (mm Hg)	68 ± 11	82 ± 12	<0.0001
Central venous pressure (mm Hg)	10 ± 4	12 ± 6	<0.01
Intra-abdominal pressure (mm Hg)	8 ± 3	8 ± 3	0.33
Abdominal perfusion pressure (mm Hg)	61 ± 11	74 ± 13	<0.0001
Cardiac index (L/min/m^2^)	2.6 ± 0.5	3.3 ± 1.0	<0.01
Respiratory pulse pressure variation (%)	10 ± 6	7 ± 3	<0.02
Arterial lactate (mmol/L)	2.8 ± 2.2	2.7 ± 2.3	0.29
Central venous oxygen saturation (%)	72 ± 7	74 ± 9	0.21
Central venous arterial PCO_2_ (mm Hg)	6 ± 2	5 ± 2	<0.05
Central-peripheral temperature (°C)	5.0 ± 1.9	5.1 ± 2.2	0.78
Sublingual microcirculation			
Total vascular density (mm/mm^2^)	16.4 ± 1.8	16.8 ± 1.0	0.19
Perfused vascular density (mm/mm^2^)	15.2 ± 2.9	16.1 ± 1.2	0.08
Proportion of perfused vessels	0.92 ± 0.14	0.96 ± 0.05	0.23
Microvascular flow index	2.6 ± 0.5	2.8 ± 0.2	0.12
Red blood cell velocity (μm/s)	912 ± 270	1,064 ± 200	<0.002
Heterogeneity flow index	1.2 ± 0.6	1.0 ± 0.2	0.09
CV red blood cell velocity	0.49 ± 0.24	0.40 ± 0.15	<0.03
Intestinal microcirculation			
Total vascular density (mm/mm^2^)	16.9 ± 1.8	17.4 ± 1.9	0.22
Perfused vascular density (mm/mm^2^)	12.3 ± 6.7	13.0 ± 6.7	0.25
Proportion of perfused vessels	0.73 ± 0.39	0.73 ± 0.37	0.88
Microvascular flow index	2.0 ± 1.1	2.1 ± 1.1	0.34
Red blood cell velocity (μm/s)	679 ± 379	747 ± 419	0.12
Heterogeneity flow index	8.9 ± 18.0	15.4 ± 42.7	0.28
CV red blood cell velocity	0.46 ± 0.36	0.37 ± 0.26	0.31

The intestinal PVD was not correlated with either the intra-abdominal pressure (*r* = 0.06, *P* = 0.81) or abdominal perfusion pressure (*r* = 0.28, *P* = 0.20).

### Correlation between sublingual and intestinal microcirculation

We observed no significant correlation between the sublingual and intestinal RBC velocities or between either the basal values or their changes in response to the fluid challenge (Figure 1). The same pattern was observed with the PVD data (Figure 2). Most of the correlations between the other microcirculatory variables had a similar pattern (Additional file 1: Figures S1, S2, S3).

On the second day, 7 patients could not be evaluated because they were either discharged from the ICU (*n* = 4) or had died (*n* = 3). In the remaining 15 patients, we observed no significant correlation between the sublingual and the intestinal PVDs, the total vascular densities, the proportions of perfused vessels, the MFIs, or the RBC velocities (*r* = 0.19, 0.15, 0.04, 0.00, and 0.02, respectively).

### Correlation of sublingual and intestinal microcirculation with other variables of tissue perfusion

The PVD and ΔT° values were not significantly correlated either at the baseline (*r* = 0.22, *P* = 0.34) or on the second day (*r* = 0.32, *P* = 0.28), but the changes in response to fluid input of these two parameters were (*r* = 0.54, *P* < 0.05). The correlation between intestinal PVD and ΔT° values were furthermore nonsignificant (Additional file 1).

### Differences between survivors and nonsurvivors

**Figure 3:**
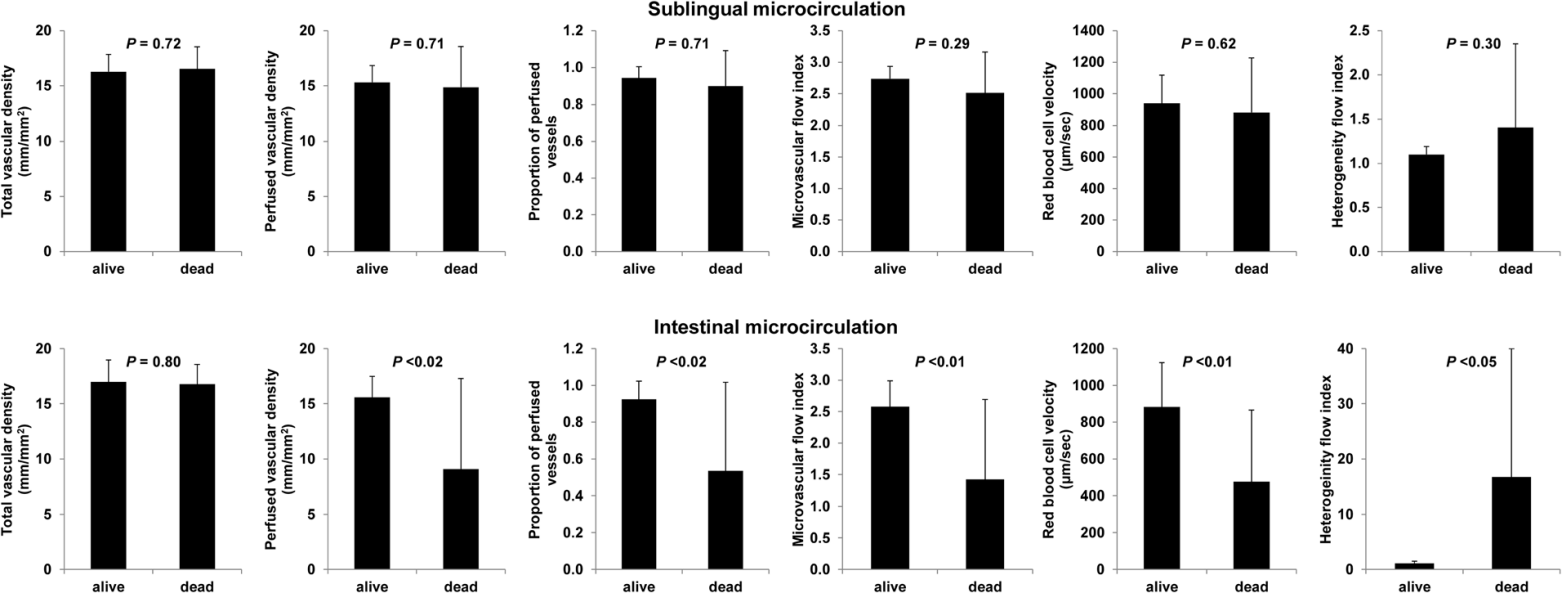
Sublingual and intestinal microcirculatory variables in survivor and nonsurvivor patients.

We found no difference in the basal sublingual-microcirculatory variables between the survivors and the nonsurvivors, whereas the intestinal parameters became more altered in the nonsurvivors (Figure 3). The ΔT° and lactate values, however, were not different between the survivors and the nonsurvivors (4.8 ± 2.2 vs. 5.3 ± 1.6°C, *P* = 0.76 and 2.3 ± 1.3 vs. 3.3 ± 2.9 mmol/L, *P* = 0.34, respectively).

## Discussion

The main finding of this study in septic postsurgical patients is the dissociation between the response of sublingual and intestinal microcirculation to a fluid challenge. This discrepancy in microvascular flow was also present at baseline and in the second postoperative day. In addition, the changes in the sublingual microvascular flow and the ΔT° values after the fluid challenge were correlated, even though the conditions of measurements of the two parameters at baseline and on the second day were unrelated. To our knowledge, the present investigation is the first clinical study that has compared the behavior of sublingual, intestinal, and skin tissue perfusion.

Experimental research on septic shock has previously assessed the behavior of sublingual and gut microcirculation during fluid resuscitation. Two studies showed that villi hypoperfusion remained present regardless of the normalization of sublingual microcirculation [10],[11]. In contradistinction, in a hyperdynamic model of cholangitis, similar abnormalities in sublingual and intestinal microcirculation were found [12]. An explanation for these contradictory findings is that in the latter study, the alteration in microvascular perfusion was extreme, with fewer than 30% of the small vessels remaining perfused. Consequently, such a severe condition might have affected the different microcirculatory territories more uniformly. Another investigation also found that sublingual and gut microcirculation displayed a similar behavior during a hypodynamic endotoxemic shock, in which cardiac output was about 50% of baseline [21]. These findings are almost identical to those of the other studies before the aggressive fluid resuscitation [10],[11] and were predictable because during states of low cardiac output, hypoperfusion is ubiquitous. Only one clinical study evaluated this specific problem: That research, performed in patients with abdominal sepsis after repeated volume administration, found a lack of correlation between the sublingual and intestinal MFIs, during the first postoperative day. This dissociation disappeared on the third postoperative day [13]. The relevant limitations of that study, however, were the lack of any basal measurements before the administration of fluids in addition to a microcirculatory evaluation that was restricted solely to the MFI. Our findings confirmed and expanded those previous results. We performed both a comprehensive assessment and an analysis of the microcirculation; moreover, we undertook the measurements at baseline, directly after the administration of solutions and on the following day. This approach allowed the identification of differences in the static characteristics of the microcirculation and also in the response to a change in the systemic hemodynamics.

The fluid challenge used here allowed us to assess the ability of increases in cardiac output to recruit the microcirculation within each microvascular compartment. The hemodynamic coherence between systemic and microvascular flow proved to be present in the tongue but not in the gut. Even though sublingual microcirculatory variables did not correlate with the cardiac output at baseline, the individual changes in that parameter were dependent on those in cardiac output. As previously reported [22], fluid challenge improved sublingual microcirculation in preload-responsive patients. Another study also showed that fluids improved the sublingual microcirculation during early septic shock [23]. Although microcirculation could behave differently from systemic hemodynamics [2], our findings suggest that the microcirculatory beds do not constitute a completely independent compartment within the cardiovascular system. Thus, therapeutic approaches aimed at improving systemic hemodynamics may also affect sublingual microcirculation in different ways. In contrast, the villi microcirculation behaved as an isolated territory that was not influenced by the changes in cardiac output.

In the entire group, the microcirculatory variables that increased after the fluid challenge were the RBC velocity and the corresponding coefficient of variation. The RBC velocity reflects microvascular convective oxygen transport. The relevance of fluid-induced changes in the RBC velocity is uncertain. Fluids might produce tissue edema, which condition could increase diffusional distances with the subsequent reduction in PVD and diffusional oxygen transport [24]. In patients with septic shock, a significant difference in sublingual PVD and heterogeneity has been reported between survivors and nonsurvivors, but the RBC velocity was similar in both of those patient outcomes [2]. These results suggest that diffusional determinants are more critical to tissue oxygenation than convective parameters. In the present study, the increase in RBC velocity occurred in the face of an unchanged PVD, suggesting that fluid challenge actually increased microvascular oxygen transport. Moreover, in fluid-responsive patients, sublingual PVD and RBC velocity improved in parallel with the increase in cardiac output. In addition, the reduction in the coefficient of variation associated with the RBC velocity could have contributed an improvement of the tissue oxygenation because of the critical role of heterogeneity in oxygen extraction [25]. Although most of the microvascular variables behaved similarly, our analysis and the present discussion are based mainly on the PVD. This parameter is the microcirculatory variable that best summarizes the convective and diffusional components of microvascular oxygen transport. The PVD reflects primarily diffusional influences, because the vascular density determines the diffusional distances along with the surface area available for oxygen exchange. In addition, PVD takes into account a convective component, such as the presence of continuous flow.

We found that a fluid-mediated improvement in sublingual microcirculation was strongly dependent on the magnitude of the microvascular disorder - the more severe the microcirculatory alteration present, the higher the improvement in response to fluids. Another study also showed that the beneficial effect of fluids was restricted to patients with a sublingual MFI lower than 2.6 [8]. That patients with more severe alterations might have greater improvement in microcirculation parameters in response to a fluid challenge would in fact be expected from a physiologic standpoint. If microcirculation is practically normal, a little room for further increase is available.

As had been previously reported [26], we found no correlation between the basal sublingual PVD and the ΔT°. In spite of this absence, the changes in these parameters in response to volume expansion were correlated. While a prediction of the state of sublingual microvascular flow from the skin perfusion was not possible, both territories behaved similarly when the preload and the cardiac output were increased. These findings also evidenced that the dissociation of the two microvascular beds is a dynamic process and not necessarily permanent.

The dissociation of territories was also expressed in the prognostic power. Indeed, the intestinal microcirculation values were more severely compromised in nonsurvivors compared to survivors, but neither of the sublingual parameters or the ΔT° was. Nonetheless, these results do not challenge the established value of sublingual microcirculation as a prognostic index in septic patients [1]-[5] but simply reflect that, in these surgical patients, the local ischemia in the villi is probably more relevant than the state of perfusion in other vascular beds. Accordingly, isolated villi ischemia might influence the outcome in the absence of microvascular disorders in other territories because of the putative role of that vascular bed in the development of multiorgan failure [9]. Furthermore, the failure of sublingual microcirculation to predict an outcome might be explained by the near normal sublingual PVD. Actually, sublingual microcirculation after the fluid challenge reached the pattern of septic shock survivors [2].

This study has limitations. The most relevant limitation is that local phenomena related to surgery could have modified the intestinal microcirculation. Although no correlation was evident between the intra-abdominal pressure and the abdominal perfusion pressure with the intestinal PVD, we cannot rule out that the effects of the surgery undertaken have compromised the vascular supply of the villi. This possibility might provide an explanation for the differing behavior of sublingual and intestinal microcirculation. Under such conditions, our results could not be extrapolated to other groups of septic patients. Nevertheless, our findings emphasize that in this group of postoperative patients, the villi microcirculation had not only a different physiologic behavior but also a higher prognostic implication. In addition, we admit that the lack of a control group with surgical stomas but without sepsis is another relevant limitation. Finally, the characteristics of the study were merely descriptive since the pathophysiologic processes involved in microvascular flow redistribution were not explored.

## Conclusions

After examining the results of the study, the following conclusions were drawn.

1) In this series of postoperative septic patients, we found a significant dissociation among the microcirculatory territories examined in both the basal condition and the response to fluid challenge.

2) Our results offer an integrative perspective of the determinants of sublingual microcirculatory response to a fluid challenge - one that is dependent on both the change in cardiac output and the basal state of the microcirculation. Therefore, we can expect a higher improvement when cardiac output is substantially increased in patients with severe microvascular derangements. Since local conditions might preclude intestinal microvascular reactivity, the behavior of sublingual microcirculation may be a better approach to the evaluation of general microcirculatory fluid responsiveness.

3) The disorder in villi microcirculation seems more related to outcome than the status of sublingual microcirculation in this series of patients with abdominal surgery.

## Abbreviations

ΔT°: gradient between the central and second finger pad temperature

ICU: intensive care unit

MFI: microvascular flow index

PVD: perfused vascular density

RBC: red blood cell

SDF: sidestream dark field

## Competing interests

Dr Ince has developed SDF imaging and is listed as inventor on related patents commercialized by MicroVision Medical (MVM) under a license from the Academic Medical Center (AMC). He has been a consultant for MVM in the past but has not been involved with this company for more than 5 years now, except that he still holds shares. Braedius Medical, a company owned by a relative of Dr Ince, has developed and designed a handheld microscope called CytoCam-IDF imaging. Dr Ince has no financial relation with Braedius Medical of any sort - i.e., he never owned shares or received consultancy or speaker fees from Braedius Medical. This work was supported by the grant PICT-2010-0495, Agencia Nacional de Promoción Científica y Tecnológica, Argentina. All other authors declare that they have no competing interests.

## Authors’ contributions

NN, LP, ARV, and NPR collected the data. AD carried out the microcirculatory measurements, performed the statistical analyses, and drafted the manuscript. VKE performed the microcirculatory analyses. VKE, CI, and AD participated in the study design and interpretation of the data. All authors read and approved the final manuscript.

## Additional file

## Supplementary Material

Additional file 1: **Supporting data.** Correlation of the microcirculation with other variables of tissue perfusion and behavior of the sublingual and intestinal total vascular density, proportion of perfused vessels, and microvascular flow index. Click here for file
