# Supplementary material for: Dissociation between sublingual and gut microcirculation in the response to a fluid challenge in postoperative patients with abdominal sepsis
Source: Ann Intensive Care. 2014 Dec 4;4:39. doi: 10.1186/s13613-014-0039-3 (PMC4298674; doi:10.1186/s13613-014-0039-3)

Dissociation between sublingual and gut microcirculation in the response to a fluid challenge in postoperative patients with abdominal sepsis

Vanina Siham Kanoore Edul[1,2], vaninaedul@gmail.com

Can Ince[1], c.ince@amc.uva.nl

Noelia Navarro[3], ncn_1979@hotmail.com

Luciana Previgliano[3], luciana_previgliano@hotmail.com

Alejandro Risso-Vazquez[3], arisso35@yahoo.com.ar

Paolo Nahuel Rubatto[3], nahurubatto@hotmail.com

Arnaldo Dubin[2,3], arnaldodubin@gmail.com

[1]Academic Medical Center, Department of Translational Physiology, Amsterdam. Netherlands

[2]Facultad de Ciencias Médicas, Universidad Nacional de La Plata, Cátedra de Farmacología Aplicada, La Plata, Argentina

[3]Sanatorio Otamendi y Miroli, Servicio de Terapia Intensiva, Buenos Aires, Argentina

This study was performed in Servicio de Terapia Intensiva, Sanatorio Otamendi y Miroli, Buenos Aires, Argentina.

Correspondence to:

Arnaldo Dubin

Direccion: calle 42 Nº 577 (1900) La Plata, Argentina

Teléfono: +5491150102431

E mail: arnaldodubin@gmail.com

Correlation of sublingual microcirculation with other variables of tissue perfusion: The PVD and ΔT° values were not significantly correlated either at the baseline (r = 0.22, *P* = 0.34) or on the second day (r = 0.32, *P* = 0.28), but the changes in response to fluid input of these two parameters were (r = 0.54, *P* <0.05). A significant correlation was found, moreover, between the sublingual PVD and the lactate levels at baseline (r = -0.62, *P* <0.01) as well as on the second day (r = –0.52, *P* <0.05), but not between the changes in those variables in response to fluid challenge (r = 0.03, *P* = 0.89).

The intestinal PVD and ΔT° values were not significantly correlated, either at baseline (r = –0.25, *P* = 0.29), in the second day (r = –0.38, *P* = 0.20), or in response to fluid loading (r = 0.12, *P* = 0.61). A significant correlation was observed, however, between the intestinal PVD and the lactate values at baseline (r = –0.57, *P* <0.01) and on the second day (r = –0.71, *P* <0.01), but not between responses of those parameters to volume expansion (r = 0.14, *P* = 0.53).

Figure 1. Figure 1. Total vascular density (TVD). Panel A. Correlation between basal sublingual and intestinal TVD. Panel B. Correlation between the changes in sublingual and intestinal TVD in response to the fluid challenge. Panel C. Correlation between the changes in cardiac index and sublingual TVD in response to the fluid challenge. Panel D. Correlation between the changes in sublingual TVD in response to the fluid challenge and the basal sublingual TVD. Panel E. Correlation between the changes in cardiac index and intestinal TVD in response to the fluid challenge. Panel F. Correlation between the changes in intestinal TVD in response to the fluid challenge and the basal intestinal TVD.

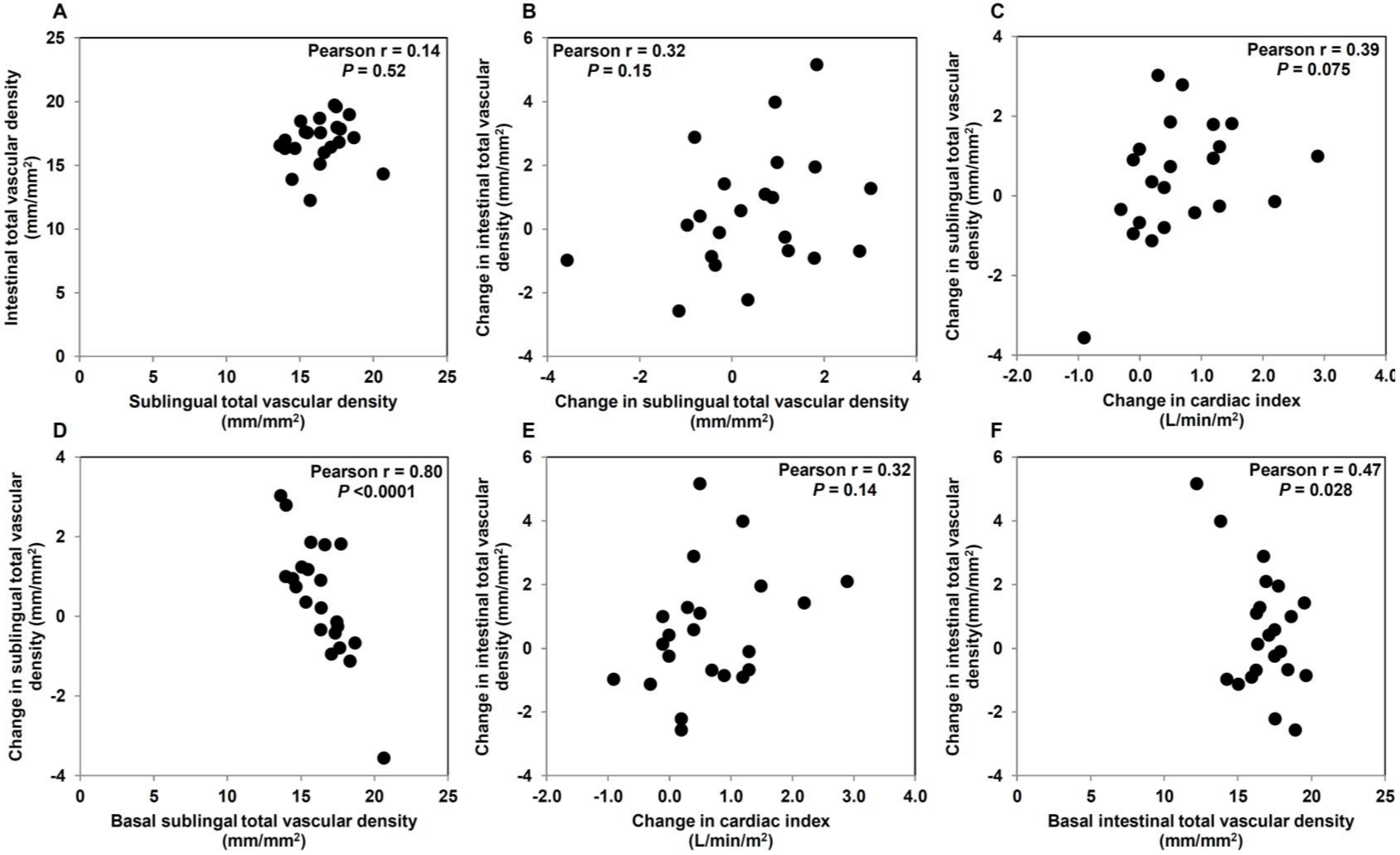

Figure 2. Proportion of perfused vessels (PPV). Panel A. Correlation between basal sublingual and intestinal PPV. Panel B. Correlation between the changes in sublingual and intestinal PPV in response to the fluid challenge. Panel C. Correlation between the changes in cardiac index and sublingual PPV in response to the fluid challenge. Panel D. Correlation between the changes in sublingual PPV in response to the fluid challenge and the basal sublingual PPV. Panel E. Correlation between the changes in cardiac index and intestinal PPV in response to the fluid challenge. Panel F. Correlation between the changes in intestinal PPV in response to the fluid c hallenge and the basal intestinal PPV.

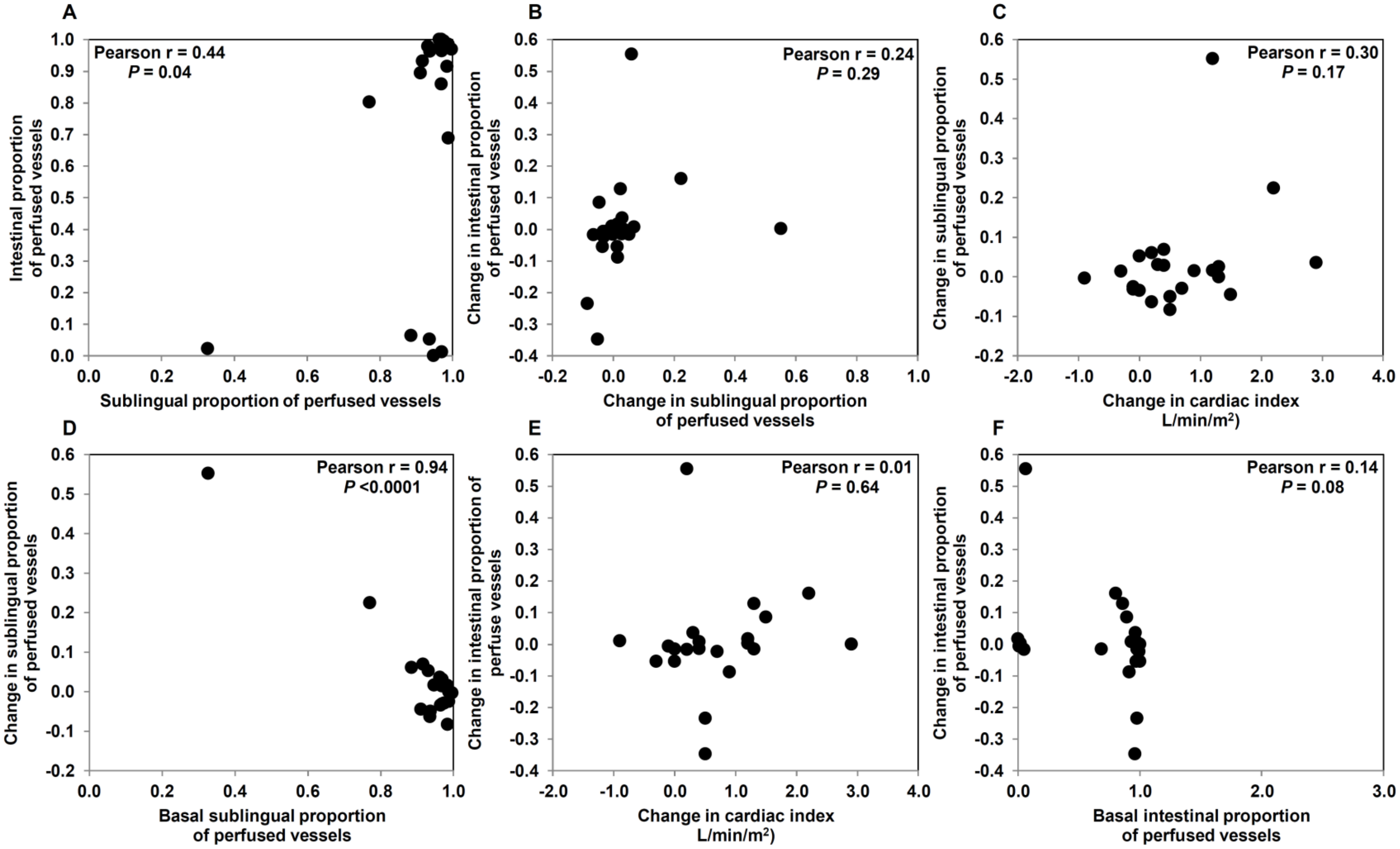

Figure 3. Microvascular flow index (MFI). Panel A. Correlation between basal sublingual and intestinal MFI. Panel B. Correlation between the changes in sublingual and intestinal PPV in response to the fluid challenge. Panel C. Correlation between the changes in cardiac index and sublingual MFI in response to the fluid challenge. Panel D. Correlation between the changes in sublingual MFI in response to the fluid challenge and the basal sublingual MFI. Panel E. Correlation between the changes in cardiac index and intestinal MFI in response to the fluid challenge. Panel F. Correlation between the changes in intestinal MFI in response to the fluid challenge and the basal intestinal MFI.

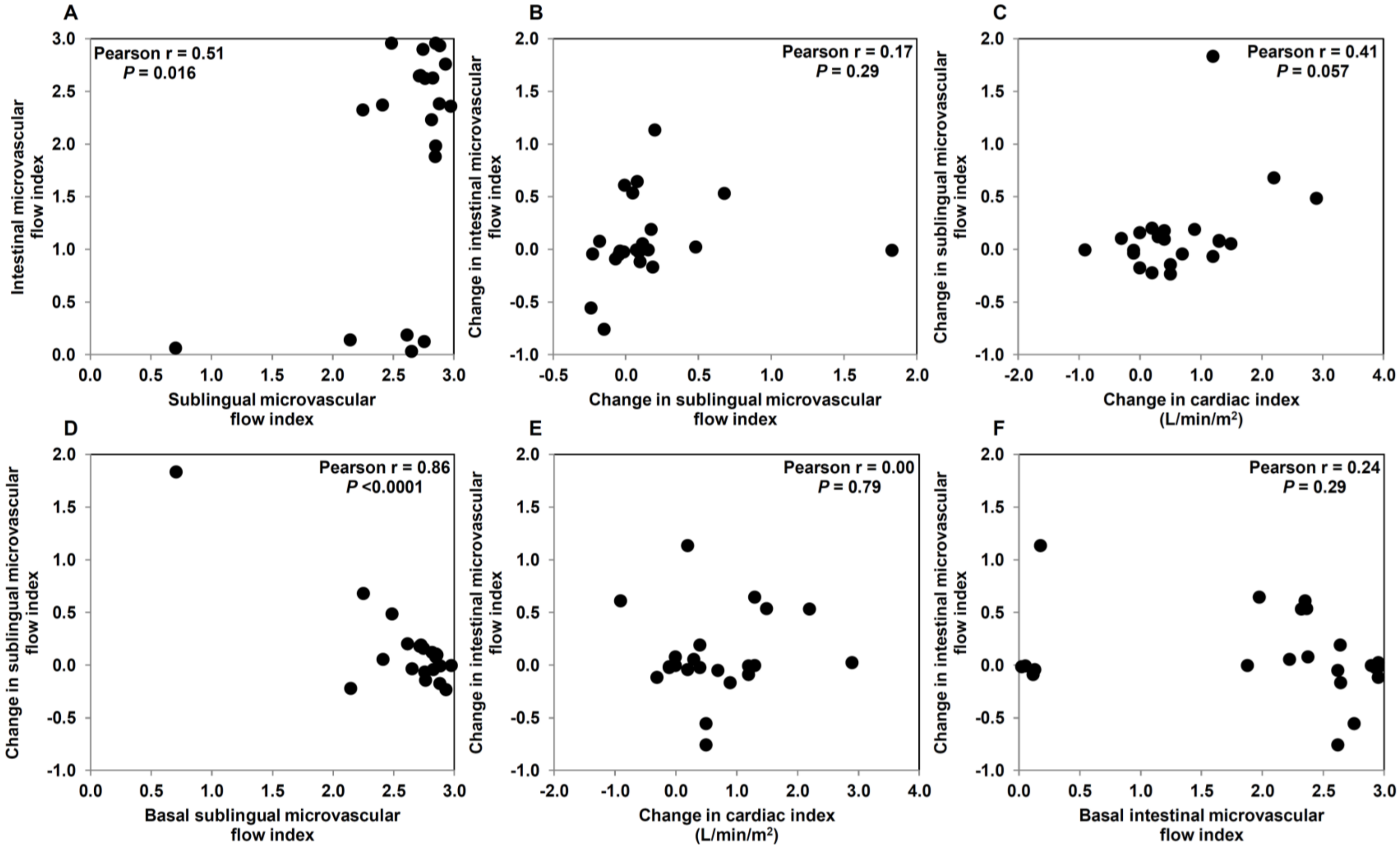

Supplement: Additional file 1: — Supporting data. Correlation of the microcirculation with other variables of tissue perfusion and behavior of the sublingual and intestinal total vascular density, proportion of perfused vessels, and microvascular flow index. [file s13613-014-0039-3-S1.pdf]
